# *AIRE* gene mutation predisposing chronic mucocutaneous candidiasis and pigmented retinitis in two kids from a Chinese family

**DOI:** 10.1080/22221751.2022.2090860

**Published:** 2022-06-28

**Authors:** Yubo Ma, Xiaowen Wang, Ruoyu Li

**Affiliations:** a Department of Dermatology and Venerology, Peking University First Hospital, Beijing, People’s Republic of China; b Research Center for Medical Mycology, Peking University, Beijing, People’s Republic of China; c Beijing Key Laboratory of Molecular Diagnosis on Dermatoses, Beijing, People’s Republic of China; d National Clinical Research Center for Skin and Immune Diseases, Beijing, People’s Republic of China

**Keywords:** *AIRE* mutation, chronic mucocutaneous candidiasis, pigmented retinitis, *Candida albicans*, whole-exome sequencing

**Figure 1.**
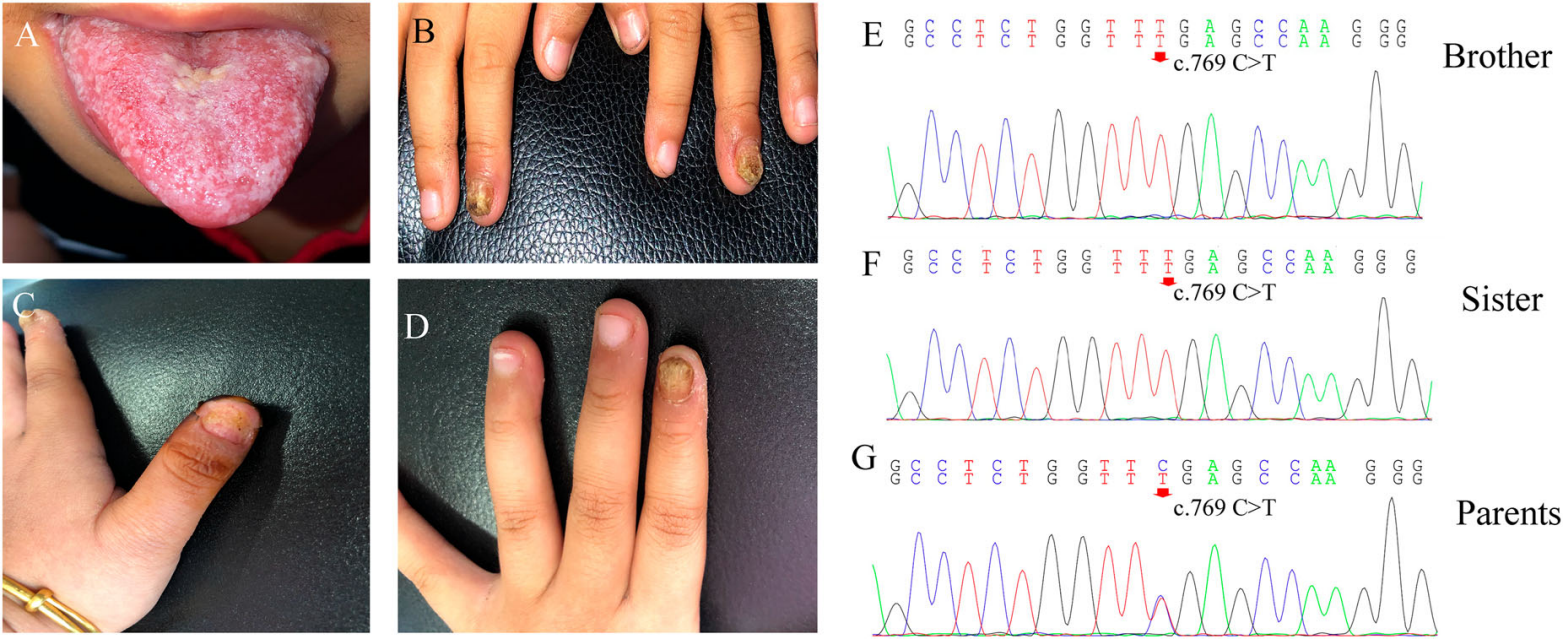
Clinical features and *AIRE* mutations in two Chinese patients with chronic mucocutaneous candidiasis. A-B. Thrush and fingernail changes of the brother. C-D. Fingernail involvement of the sister causes shedding of the nail plate and oozing scabs of the nail bed. E-F. DNA sequencing chromatograms demonstrate homozygous *AIRE* mutation in exon6: c769 C > T, p. Arg257Ter in the brother and sister. G. The heterozygous carriers of the variant in exon6: c769 C > T of their parents.

A 2-year-old boy was admitted to the hospital because of recurrent thrush since the age of 6 months, along with yellowish discoloration and thickening of his nails. Physical examination revealed oral mucosal erosion and white patches on the tongue with a pseudomembrane (Figure 1(A)). Two of his fingernails were thick and discolored with erythema around (Figure 1(B)). He also experienced progressively impaired vision, which was diagnosed as retinitis pigmentosa, causing no light perception in one eye and light perception up to 0.1 in the other. His 4-year-old sister had similar dermatological manifestations with 3 years of recurrent thrush and fingernail involvement (Figure 1(C, D)). Direct microscopic examination of their nails and the brother’s tongue showed branched pseudohyphae and yeast cells, and *Candida albicans* was identified through fungal culture. The rest of the physical examinations did not reveal any other obvious abnormalities. Their parents belonged to the *Hui* population (a minority population in China) and had a history of consanguineous marriage. Their father had a history of onychomycosis with unknown specific pathogens. Chronic mucocutaneous candidiasis (CMC) was diagnosed, and oral fluconazole was prescribed at a daily dose of 150 mg for the brother and 200 mg for the sister. After continuous fluconazole treatment for 6 months, the nails and the tongue became normal. These patients are still under follow-up.

Due to the recurrent *Candida* infections and history of consanguineous marriage, genetic susceptibility was suspected. Genomic DNA of all family members was extracted from peripheral blood leukocytes, and whole-exome sequencing (WES) was performed. When we compared the data with all genes reported to be related to CMC, a homozygous mutation in the *AIRE* gene was noted (c. 769 C > T, p. Arg257Ter) in both patients (Figure 1 (E, F)). The parents were heterozygous carriers of the variant (Figure 1 (G)).

CMC is a unique type of candidiasis that manifests as severe and recurrent or persistent infections of the skin, nails, and mucosa by *Candida* species. In the past decade, various gene mutations, including *STAT1*, *STAT3*, *AIRE, CLEC7A, CARD9, RORC, ACT1, IL-17RA, IL-17RC,* and *IL-17F*, have been reported to predispose individuals to CMC [1]. *STAT1* mutations among these mutations are considered the most common cause of isolated CMC worldwide, whereas mutations in the *AIRE* gene usually cause a syndrome named autoimmune polyendocrinopathy candidiasis and ectodermal dystrophy (APECED) mostly in northern European Caucasians [2–4]. Clinical manifestations of APECED vary greatly, usually starting in infancy with CMC, followed by autoimmune attacks on the parathyroid glands, adrenal cortex, thyroid, and other organs, including the retina [5]. The deficiency of *AIRE* impairs central immune tolerance, generating pathogenic autoreactive T cells and autoantibodies directed against many specific cytokines, such as IL-17 and IL-22 [6]. Pigmented retinitis is considered as one of the APECED manifestations, affecting 3.6% of APECED patients [7, 8]. It destroys the visual field in the early stages and affects vision, leading to blindness in the later stages [9, 10]. Patients visiting our clinic with *AIRE* mutations presented only CMC and pigmented retinitis, whereas other manifestations of APECED might still appear with age and should be closely monitored and followed up. These patients remind us of the importance of genetic analysis, which helps to adjust the time of treatment, as well as predict and detect related complications early in CMC management.
